# Conical ring array detector for large depth of field photoacoustic macroscopy

**DOI:** 10.1364/BOE.386585

**Published:** 2020-04-09

**Authors:** Paul R. Torke, Robert Nuster, Guenther Paltauf

**Affiliations:** Department of Physics, University of Graz, Austria

## Abstract

Photoacoustic microscopy and macroscopy (PAM) using focused detector scanning are emerging imaging methods for biological tissue, providing high resolution and high sensitivity for structures with optical absorption contrast. However, achieving a constant lateral resolution over a large depth of field for deeply penetrating photoacoustic macroscopy is still a challenge. In this work, a detector design for scanning photoacoustic macroscopy is presented. Based on simulation results, a sensor array geometry is developed and fabricated that consists of concentric ring elements made of polyvinylidene fluoride (PVDF) film in a geometry that combines a centered planar ring with several inclined outer ring elements. The reconstruction algorithm, which uses dynamic focusing and coherence weighting, is explained and its capability to reduce artefacts occurring for single element conical sensors is demonstrated. Several phantoms are manufactured to evaluate the performance of the array in experimental measurements. The sensor array provides a constant axial and lateral resolution of 95 µm and 285 µm, respectively, over a depth of field of 20 mm. The depth of field corresponds approximately to the maximum imaging depth in biological tissue, estimated from the sensitivity of the array. With its ability to achieve the maximum resolution even with a very small scanning range, the array is believed to have applications in the imaging of limited regions of interest buried in biological tissue.

## 1. Introduction

Photoacoustic microscopy provides an alternative to established imaging methods of biological tissue, offering high resolution and high contrast for light-absorbing structures [1]. In optical resolution photoacoustic microscopy (OR-PAM), a resolution on the micrometer scale is achieved by focusing the excitation beam and scanning over the surface point by point. The strong scattering of light in biological tissue prevents high resolution by optical focusing beyond the diffusion limit [2]. Deeper imaging is possible with acoustical resolution photoacoustic microscopy (AR-PAM), which uses a wider illumination beam [3]. Here, the spatial resolution is accomplished by the focusing properties of the ultrasound transducer and can reach up to 50 µm [4]. Deeply penetrating photoacoustic imaging methods using focused detector scanning can reach an imaging depth of a few centimeters, with a lateral resolution of about 500 µm and are denoted as photoacoustic macroscopy [3,4]. Compared to photoacoustic imaging devices that use large arrays and tomographic reconstruction of absorbing structures distributed over extended volumes, the AR-PAM approach is slower owing to the pointwise scanning, but it can use smaller lasers with higher pulse repetition rates due to the smaller irradiated volume.

In applications of AR-PAM, where the depth of the actual imaging target is not known beforehand or where the observed structures are spread over the entire penetration depth, it would be beneficial to achieve high lateral resolution over a range of several millimeters to centimeters. This is, however, not possible with the usual combination of planar ultrasound sensors and lenses with spherical refracting surfaces, which suffer from the well-known trade-off between lateral resolution and depth of field (DOF). Only within the DOF, sensor-lens combinations with large aperture have favorable properties of high lateral resolution and sensitivity [5]. Outside the DOF, the signal amplitude and lateral resolution decrease rapidly. To address this limitation, reconstruction techniques have been proposed [6,7], which provide constant lateral resolution over a large depth range, provided that detector data over a sufficient scanning length are available. Alternatively, if such data are not available, single element transducers such as axicons and ring sensors provide a much larger DOF than a spherically focused sensor but have to deal with X-shaped artefacts over the entire DOF [8]. To reduce the level of artefacts, a ring array can be used, which reaches a similar DOF but reduces artefacts with the aid of dynamic focusing onto the ring axis and coherence factor weighting [9–14]. A photoacoustically excited sound wave generated on the ring axis is temporally spread when it hits the planar sensor element, which reduces its typically broad spectral bandwidth by a degree that is given by the angle of incidence and the ring width [11]. Therefore, the lateral resolution is limited by the ring widths of the sensor elements and the overall sensitivity is much lower compared to a spherical lens detector. To overcome the drawbacks of flat ring sensors and to benefit from advantages of focused detection on the one hand and annular arrays on the other hand, a combination of ring elements arranged on an inclined substrate seems to be a promising approach [15]. In this work, a new sensor design with partially inclined rings arranged on a conically shaped surface is proposed.

In the following, we present simulation results showing comparisons of the conical array with other focusing geometries, such as single spherical or conical sensors. Based on the results, we manufactured an array using a film of the piezoelectric polymer polyvinylidene fluoride (PVDF) and verified its performance in experiments on various phantoms. The results demonstrate a large DOF, a reduction of artefacts caused by dynamic focusing and a good lateral resolution over a wide range of depths.

## 2. Array design

### 2.1 Simulation method

To find a suitable sensor geometry, we performed a simulation study using methods that we earlier used for axicon [8] and planar ring array sensors [11]. In the simulations, the response of an ideal, broad-bandwidth sensor with finite area is calculated to a distribution of photoacoustic sources, arranged at different depths. The initial pressure *p*_0_ of a photoacoustic source at an arbitrary point $\text{r}\in R^{3}$ can be described as the product of the absorbed optical energy density distribution W(r) in the medium and the Grueneisen parameter Γ [16].

(1) $$p_{0}(\text{r})=\Gamma W(\text{r})$$

In a lossless medium, where the attenuation of the ultrasound wave can be neglected and a short laser pulse with temporal function I(t) is used for illumination, the photoacoustic wave equation can be expressed as [17]

(2) $$\left(\nabla^{2}-\frac{1}{c_{s}^{2}}\frac{\partial^{2}}{\partial t^{2}}\right)p(\text{r},t)=-\frac{\beta }{C_{p}}W(\text{r})\frac{\partial I(t)}{\partial t}$$

Here, $c_{s}$ denotes the speed of sound, β is the thermal coefficient of volume expansion and *C_p_* the specific heat capacity at constant pressure. A solution of this equation for a specific transducer geometry can be found under two commonly used assumptions. First, the laser pulse duration is sufficiently short to express I(t) as a Dirac delta function δ(t). Second, the transducer with its finite surface can be viewed as a linear system and thus its response can be described as an integral of ideal point detector signals. From this follows the temporal signal received by a detector with the surface $S_{0}$ centered at the origin [1].

(3) $$|s(t)=\frac{\beta }{4\pi C_{p}}\frac{\partial }{\partial t}\int_{S_{0}}\int_{V}W(r)\frac{1}{\left|r_{0}-r\right|}\delta \left(t-\frac{\left|r_{0}-r\right|}{c_{s}}\right)d^{3}rdS_{0}$$

Here, ${\text{r}}_{0}$ is the position vector of a point on the detector surface $S_{0}$ and the integration goes over the volume *V* containing the source distribution W(r). The spatial impulse response for an arbitrary detector geometry can be expressed as

(4) $$h(r,t)=\int_{S}\frac{1}{\left|r_{0}-r\right|}\delta \left(t-\frac{\left|r_{0}-r\right|}{c_{s}}\right)dS_{0}$$

h(r,t) is the signal received by the detector from a point source located at r [18,19]. Since the goal of the simulation is a comparison between different sensor geometries, we neglect the prefactors of the integral in Eq. (3). Furthermore, we assume the energy density distribution as being represented by the positions and sizes of spherical photoacoustic sources located at positions ${\text{r}}_{\text{i}}$. Then, the simulated signal received by the sensor *s*(*t*) can be expressed as a temporal convolution of the typical bipolar N-shaped signal of a spherical source g(t) with the sum of spatial impulse responses of the sensor, $h({\text{r}}_{\text{i}},t)$, for the given positions ${\text{r}}_{\text{i}}$ of the spheres.

(5) $$s(t)=g(t)*\underset{i}{\sum }h({\text{r}}_{\text{i}},t)$$

**Fig. 1.**
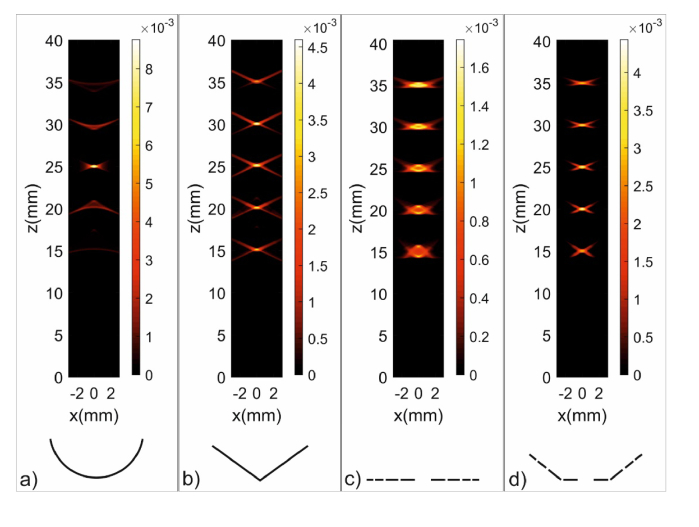
Comparison of simulated 2D images of a phantom consisting of five spherical sources with 0.5 mm diameter, arranged at depths between 15 and 35 mm: a) spherically focused detector, b) axicon, c) four-element planar ring array, d) four-element ring array with three inclined rings and a planar ring in the center.

For all simulations, we defined the size of the spherical sources with 0.5 mm diameter, the speed of sound as 1.5 mm/µs and a unit peak to peak amplitude of the bipolar N-shaped signal in g(t). To simulate a two-dimensional (B-scan) image, the distribution of sources, which were arranged along a line in z-direction, was scanned linearly (along x-direction) across the sensor. The signals obtained at each scanning position were Hilbert transformed. In the case of several sensor elements, the procedure was repeated for each element. Knowing the speed of sound, the temporal signals contain depth information of the individual sources. For a ring-shaped sensor with defined inner and outer radii, there exists a relationship between the arrival time of a signal from the ring axis and the axial position of the source. This relation can be used to interpolate the signals received by the ring sensors in the concentric array to a common *z*-axis, thereby forming a dynamic focus after adding up the interpolated signals [11]. To reduce artefacts caused by off-axis sources, the summed signals were multiplied by a coherence factor, which enhanced amplitudes if signals received by different sensors interfered constructively [20]. The arrangement of the positive parts of the amplitude (A-) scans for successive scan positions in x-direction leads to the B-scan image shown in Fig. 1.

### 2.2 Comparison of simulated detector geometries

Figure 1 shows simulated B-scan images regarding four different sensor geometries of a numerical phantom consisting of spherical sources. The purpose of this comparison is to illustrate the influence of the sensor geometries and their advantages and disadvantages. A sensor with spherical surface was simulated with a center of curvature at a depth of 25 mm. It had a diameter of 28 mm, equal to the outer diameter of the other simulated sensors. As expected, it provides optimal lateral and axial resolution for a source scanning across the focus. Due to the large numerical aperture, the DOF is too small to depict any of the sources located outside the focus. The second sensor had conical shape with an angle of 25° (relative to the plane perpendicular to the cone axis) and a diameter of 28 mm. With this axicon sensor, the axial and lateral widths of all five spheres are almost equal in the image. Moreover, the lateral widths are similar but slightly larger than with the spherical sensor. However, strong X-shaped artefacts are clearly visible over the entire DOF. Such X-shaped structures are typical for ultrasonic axicon transducers and are related to the phenomenon of “X-waves” generated by pulsed excitation [21,22]. Although they seem to be a minor problem for these defined and well-separated sources, they can lead to ambiguities in images of more complex structures. The third sensor was a planar ring array consisting of four concentric rings with equal area, which was achieved by defining the innermost ring with an inner radius of 3 mm and outer radius of 7 mm. The sizes of the other ring elements were subsequently found by ensuring the same area as the inner ring element, yielding an outermost radius of 28 mm. By applying dynamic focusing and coherence factor weighting, the artefacts are strongly reduced. However, both the axial and lateral resolution are low, owing to the width of the elements, which can be improved by using thinner ring elements [11]. Smaller ring widths, on the other hand, do not utilize the available transducer area, limiting the sensitivity of the device.

The proposed geometry consisting of one flat and three inclined annular ring elements combines good lateral resolution and sensitivity over a large DOF. It benefits from dynamic focusing and coherence weighting, leading to images with low level of artefacts. The sizes of the ring elements were defined similar to the previously described ring array, whereby the conical shape with an angle of 25° was considered when the area of the inclined ring elements was calculated. In comparison to the flat array with an equal number of rings and the same ring areas, the signal amplitude is increased almost threefold and the lateral resolution is significantly higher, comparable to the value obtained with the conical sensor.

## 3. Methods

### 3.1 Sensor array design and manufacturing

**Fig. 2.**
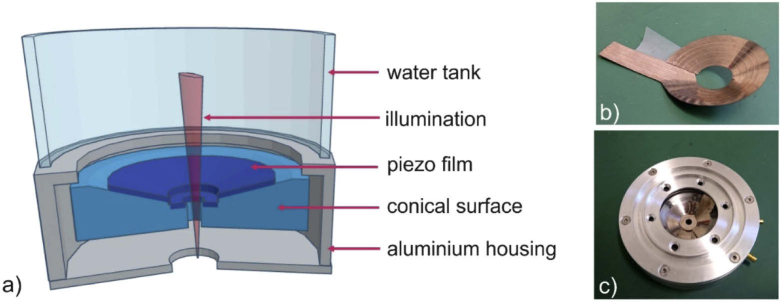
Schematic illustration and photographs of the presented sensor array: a) cross section image of the geometry of the sensor array, b) photograph of the lower side of the piezo film, showing the etched elements, c) photograph of the device with the aluminium housing containing the plugs for the electrical connection to the amplifier.

A five-ring array was constructed with a geometry that is schematically illustrated in Fig. 2(a). The sensor array consists of two pieces of a 110 µm thick PVDF film, which was metallized on both sides. The detector array contained four outer inclined rings and one flat ring in the center. The flat ring element in the center of the sensor array ensures the near-vertical incidence of ultrasound waves from sources close to the sensor surface. The patterning of the PVDF film with the four elements was performed by electrically etching the lower conductive layer with a needle and a power supply. When the needle touched the conductive layer, an electric circuit was closed and the generated current evaporated the metal. The etching needle was steered with the aid of a modified 3D printer (Velleman, K8200), which had a minimum step size of 250 µm. The conductive layer was removed along concentric lines with radii of 7.75, 10.75, 13.25, 15.25 and 17.25 millimeters, which lead to ring-shaped electrodes with similar active areas. In addition, for each annular element a radial connecting line was etched onto the film to enable contact to external electrodes. The distance between the conducting areas was 500 µm, corresponding to the mean diameter of the etching area of the needle. The photograph of Fig. 2(b) shows the lower side of this piezo film, displaying the etched ring elements. The lower conductive layer of the inner, flat ring was completely etched away with aid of iron chloride (FeCl_3_) solution. The lower electrode was then formed by gluing the film onto a copper ring. All oblique rings were attached onto a conical surface with an angle of 25 degrees. The central hole through the flat ring enabled illumination of the samples and was sealed with a glass slide. The upper conducting layers of both PVDF pieces were electrically connected with a silver conductive varnish and formed the common ground via contact with the aluminum housing of the detector array, which is shown in Fig. 2(c). To guarantee acoustic coupling between the sample and the ring array, a water tank was placed on top of the detector housing. The electrical contact to the central electrode was provided by a socket plug in the copper ring. Four cables were separately glued with conductive epoxy to the radial lines on the lower side of the inclined PVDF film. All cables were connected to plugs in the housing wall of the detector array.

### 3.2 Imaging setup

**Fig. 3.**
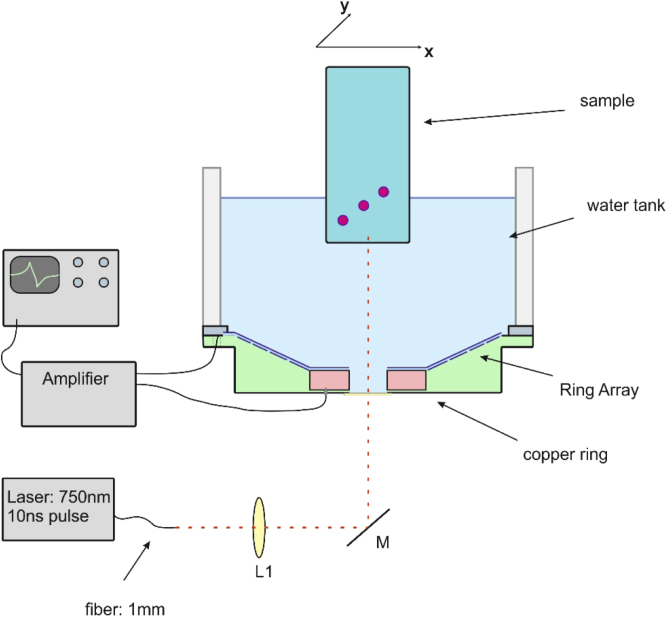
Schematic drawing of the sensor array and the measurement setup.

A schematic drawing of the imaging setup is shown in Fig. 3. The sample was illuminated with pulses from an optical parametric oscillator (OPO) tuned to 750 nm wavelength, which was pumped by the second harmonic of a Q-switched Nd:YAG laser. For all measurements, the pulse duration was 6 ns and the pulse repetition rate was 20 Hz. The radiant exposure was separately determined for each phantom.

The laser light was guided through a 1 mm core diameter optical fiber. The fiber end face was imaged with about six-fold magnification onto the surface of the sample. All signals were amplified with a home-made amplifier, having a bandwidth from 80 kHz to 36 MHz at -3 dB and a gain of 58.6 dB at 1 MHz. An 8-bit, four channel oscilloscope was used for simultaneous data acquisition, with a low pass filter of 20 MHz cut-off frequency to reduce high frequency noise. Electrical crosstalk between the rings could not be observed and was therefore assumed as negligible. The whole device acted as a broadband transducer and had an ultrasound bandwidth from 0.5 MHz to 10 MHz, which was estimated from the response to a 110 µm diameter microsphere placed at a depth where the generated ultrasound wave arrived at normal incidence to an inclined ring and with 100 times signal averaging. For all further experiments, signals were measured without averaging. Scanning the sample in x- and y-direction was accomplished with the aid of two motorized stages.

### 3.3 Reconstruction of A-scans

Using the definition of h(r,t) in Eq. (4), the response of the sensor elements to a small, spherical source on the line of sight (i.e. the symmetry axis) of the sensor array was calculated with

(6) $$M({\text{r}}_{\text{c}},t)=g(t)*h({\text{r}}_{\text{c}},t)$$

where ${\text{r}}_{\text{c}}$ denotes a point on the axis. The function g(t) contains the N-shaped signal from a homogeneously irradiated small sphere with a size of 50 µm, which is below the resolution of the device. After discretization in space and time, *M* becomes a two-dimensional matrix. It contains the relationship between the time of flight from a source located on the ring axis and its depth. The reconstruction was performed by multiplying the normalized and transposed matrix of each ring with the corresponding measured temporal signals to obtain A-scans. Coherence weighting was applied after adding the A-scans of all rings.

### 3.4 Phantoms

Several phantoms were prepared to assess the performance of the ring array. For all experiments, the bulk material of the phantoms contained distilled water and 2% of Agarose to achieve similar acoustic properties. Depending to the task, the phantoms differed in the optical scattering coefficient of the bulk material and the type of the inserted objects as well as their absorbing properties. To make the agar optically diffuse, we used SMOFlipid (20% fat emulsion), which provides known scattering properties [23] and is comparable to known fat emulsions, such as Intralipid [24]. Absorption coefficients were determined from the optical transmittance of the phantom materials. The reduced scattering coefficient of the turbid agar was measured by oblique incidence reflectometry based on the work of Wang and Jacques [25].

Regarding the acoustic properties, we assume that SMOFlipid behaves like Intralipid (20%) due to its similar composition. Intralipid-containing phantoms have been thoroughly investigated for their acoustic properties and were found suitable for mimicking biological tissue, particularly regarding the acoustic attenuation [26].

### Microsphere phantom

To estimate the lateral and axial resolution of the sensor array, a microsphere with a diameter of 90-110 µm was embedded in a transparent agar phantom. B-scans were measured by scanning the phantom across the symmetry axis of the array at different distances.

This phantom was also used for a calibration measurement in order to correct the signals for the varying sensitivities of the ring elements, partly due to the manufacturing process and also due to the different distances to the common symmetry axis. The goal was to achieve similar response of the device to sources of equal strength along the entire DOF. For each depth position of the microsphere the maximum value of the ring with the highest amplitude was determined and the signals received by all rings from this depth were weighted with the reciprocal of this value.

### Lead phantom

**Fig. 4.**
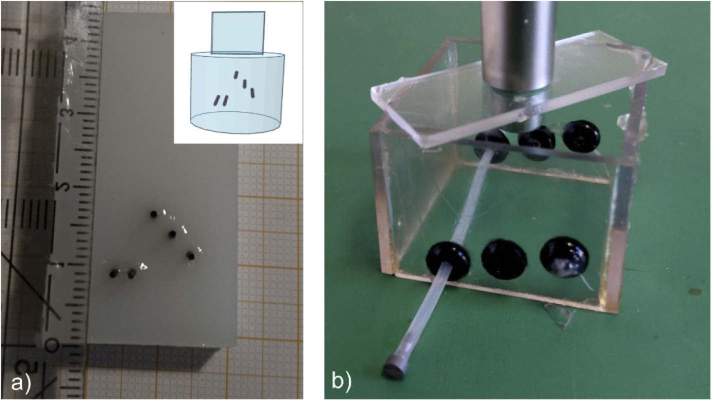
Photographs of two phantoms: a) Orientation of the graphite leads in the background material, consisting of agar and SMOFlipid, b) photograph of the tube phantom that was used to estimate the NEP of the sensor array.

Earlier work has shown that a line-shaped source scanned across a single element ring or axicon sensor gives rise to strong X-shaped artefacts [8]. The second phantom was therefore built to provoke such artefacts and to investigate to which degree they can be reduced by dynamic focusing. Several graphite leads with a diameter of 0.7 mm were embedded in agar, which was mixed with 2% SMOFlipid. The photograph in Fig. 4(a) shows the orientation of the leads in an intermediate step, before they were entirely surrounded with the background material of agar and SMOFlipid. The graphite leads were positioned parallel to each other at different depths and were displaced along the scan-axis (the x-axis). The measured reduced scattering coefficient of the carrier material was 0.41 mm^−1^ and the phantom was illuminated uniformly with 7.6 mJ over a circular area of 7 mm diameter.

### Tube phantom

Two phantoms containing tubes with absorbing liquid in a scattering bulk material were constructed. The tube phantom used for an estimation of the sensor sensitivity is shown in Fig. 4(b). It contained a single silicon tube with an inner diameter of 1 mm oriented perpendicularly to the scan direction, at a distance of 30 mm from the planar ring. The tube was filled with an ICG solution with an absorption coefficient of 2 mm^−1^. It was embedded 5 mm deep in a gel containing 2% agarose, 4% SMOFlipid and a small amount of ICG to achieve a reduced scattering coefficient of 0.78 mm^−1^ and an absorption coefficient of 0.015 mm^−1^. With a pulse energy of 5.5 mJ and an illuminated area of 0.28 cm^2^ the incident radiant exposure on the surface of the phantom was 19.6 mJ/cm^2^.

To analyze the imaging properties of more complex and less regular objects, another phantom was constructed, which allowed the insertion and repeatable replacement of various tubes. Several silicone tubes were arranged irregularly in various depths and intersecting each other, as shown in Fig. 9(a). The inner diameter of the tubes was either 0.5 or 1.0 mm. Relative to the surface of the phantom, the ends of the tubes were positioned at depths of 5.0, 7.5 and 12.5 mm in the frame. Due to the bending of the tubes between their fixed ends, their effective depth could vary between 2.5 and 15 mm. The carrier material of this phantom consisted of distilled water with 2% agarose and 1% SMOFlipid, which lead to a reduced scattering coefficient of 0.25 mm^−1^. The tubes were filled with a dye solution consisting of distilled water, 2.5% albumin and 0.025% indocyanine green (ICG) [27], with an absorption coefficient of 1.35 mm^−1^.

## 4. Results

The performance of the sensor array was investigated in three different experiments. To evaluate the performance of the sensor for objects, which are positioned farther to the sensor surface, the planar ring and three outer rings were used for all measurements. B-Scans were obtained along the x-axis with a fixed increment of 50 µm. A mean value in scan direction was subtracted from the raw B-scan data to reduce scan position independent artefacts, such as electrical interference from the pulsed laser or pyroelectric signals caused by scattered light. The reconstruction was calculated as explained in chapter (3.3).

### 4.1 Dynamic focusing and coherence factor weighting

**Fig. 5.**
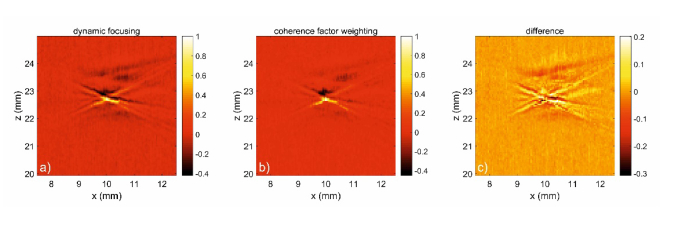
Imaging results of a microsphere phantom: a) B-scan of the phantom after dynamic focusing, b) B-scan of the phantom after dynamic focusing and coherence factor weighting, c) The difference between the two B-scans.

Figure 5 illustrates the B-scan of a microsphere phantom after dynamic focusing (a) and after additional coherence factor weighting (b). The comparison of the two B-scans shows that the contrast-to-noise ratio (CNR) is increased from 65 to 90 by coherence factor weighting while the full width at half maximum (FWHM) remains almost the same (235 µm before and 245 µm after weighting). This is consistent with the observation that the use of the coherence factor weighting does not improve the lateral resolution of an image as stated in Ref. [28]. However, with the help of the coherence factor, the x-artefacts, as well as the background noise could be reduced, which is illustrated by the difference between the two B scans shown in Fig. 5(c). We would like to emphasize that the FWHM calculated after the reconstruction method does not provide a direct information about the possible resolution based on the sensor geometry, as it was also pointed out in [28]. Since the microsphere measurements have shown that the FWHM with and without the use of coherence factor weighting hardly changes, we will in the following use the FWHM after the complete reconstruction to interpret the performance of the array.

### 4.2 Depth of field

**Fig. 6.**
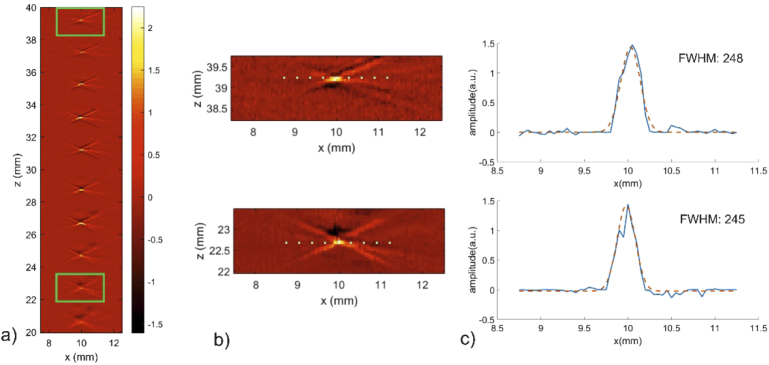
Imaging results of a microsphere phantom after reconstruction: a) B-scans of the phantom for different depths. The results are superimposed in one image, b) B-scans of the phantom at depths of about 22.5 mm and 39 mm, c) The resolution was estimated from the FWHM of a Gaussian fit (red dotted line) to profiles through the center of a microsphere (blue continuous line).

Figure 6(a) shows several reconstructed B-scans of the microsphere phantom positioned at different depths and added in one image. The widths of the temporal signals increase slightly for microsphere positions closer to the detector. This can be explained by the oblique incidence of the acoustic wave on the outer rings, which leads to some temporal spreading of the signal, similar to the results of the simulation for a planar ring array. Additionally, the planar ring of the sensor array, which does not provide a sharp focus on the axis due to its width, contributes a signal with high axial but low lateral resolution to the image of objects close to the array. The lateral and axial resolution were measured after reconstruction by fitting a Gaussian curve to profiles of the microsphere images at each depth. Taking mean values of FWHM over all measurements results in a mean lateral and axial resolution of 285 µm and 95 µm, respectively.

**Fig. 7.**
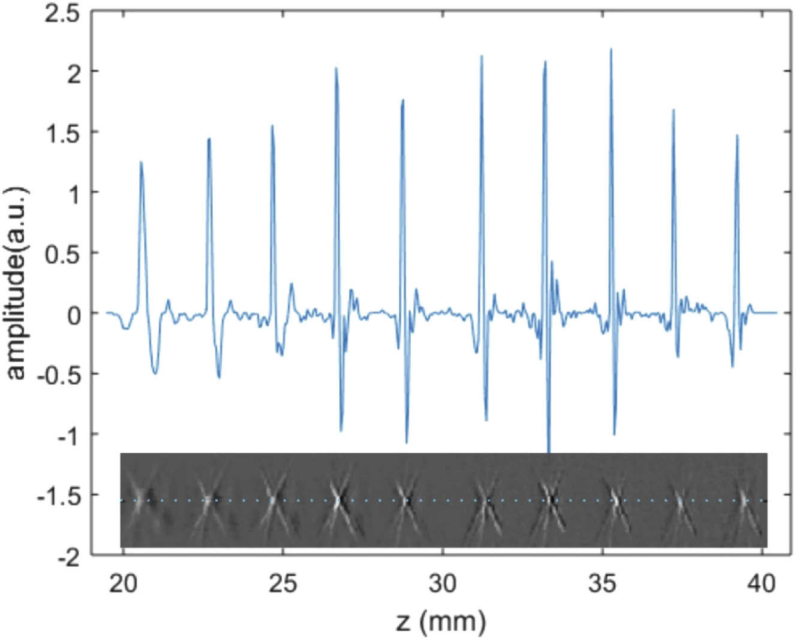
Axial profile through the center of the reconstructed microsphere image. The amplitudes of the microsphere images stay consistent for depths from 20 mm to 39 mm.

Since the microsphere was embedded in transparent agar and the illumination of the sample could be assumed as constant over the depth of field, the amplitudes of the reconstructed B-scans can be compared to each other to estimate the dependence of sensitivity of the sensor array on depth. In Fig. 7 the central profile trough the B-scans is shown over a depth range from 20 mm to 39 mm relative to the sensor surface. Owing to the calibration procedure described above, the amplitudes do not vary significantly over a range of about 20 mm.

The results of the microsphere experiment show that the sensor array exhibits a large depth of field from 20 mm up to 39 mm, where resolution and sensitivity stay approximately constant. The focusing range of almost 20 mm can be described as the part of the symmetry axis, from which spherical waves arrive at almost normal incidence at the sensor surface of at least one ring element. Accordingly, each of the rings has its optimum focusing range defined by the normal incidence of waves originating at the sensor axis.

### 4.3 Sensitivity

The single tube phantom was used to estimate the signal to noise ratio (SNR) and the noise equivalent pressure (NEP) of the sensor array. The NEP is defined here in a way that it describes the initial pressure amplitude of an object located at the sensor axis that gives a reconstructed A-scan amplitude equal to the root mean square (RMS) value of the noise. The actual pressure signal arriving at the sensor surface has a lower amplitude. The initial pressure at the target can be expressed as $p_{0}(\text{r})=\Gamma W(\text{r})$ with the Grueneisen parameter of Γ = 0.12 for the measured water temperature. Using a Monte Carlo simulation for calculation of the mean energy density distribution in the tube yielded an initial pressure $p_{0}$ = 6 kPa [19]. Analyzing the reconstructed image of the tube gave a signal to noise ratio of 105, which leads to a NEP of about 57 ± 5 Pa.

### 4.4 Artefact reduction

**Fig. 8.**
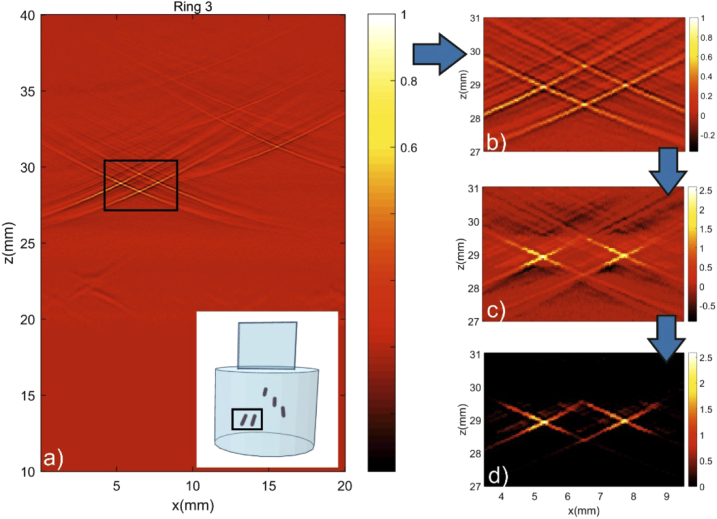
Artefact reduction based on dynamic focusing and coherence weighting: a) B-scan of ring 3 across the phantom shown in the inset, b) Zoom of the area indicated by the rectangle in a) showing two leads and illustrating the overlapping effect of X-artefacts for objects positioned next to each other, c) Reconstruction results of the two leads after dynamic focusing of all rings and the use of coherence factor weighting, d) Reconstruction without negative values.

The second phantom was developed to produce the typical X-shaped artefacts, which are a challenge for conical sensor geometries. Scanning a line-shaped object orthogonal to its direction with a conical ring sensor induces a stronger X-shaped artefact than a point like object. This is because the point source generates a spherical wave, which produces stronger constructive interference on the conical sensor surface. The effect becomes apparent in Fig. 8(a), which shows a schematic depiction of phantom 2 and the corresponding B-scan for ring 3. Figure 8(b) shows a zoom of the B-scan of two leads positioned next to each other and the effect of their overlapping X-artefacts. At the crossing points of the artefacts, the amplitude rises in a similar way as for a real source, giving rise to the observed ambiguity with four instead of two objects. Since the conical sensor is divided into several ring elements with different diameters, each individual B-scan has a different enclosed angle of the X. Adding up the individual reconstructed images and applying coherence factor weighting reduces the artefacts considerably, as it is shown in Fig. 8(c). Figure 8(d) displays the positive values of the reconstructed image. The positions of both leads are enhanced and the “ghost objects” of the previously overlapping X-shaped structures are removed.

### 4.5 Intertwined tube structures

**Fig. 9.**
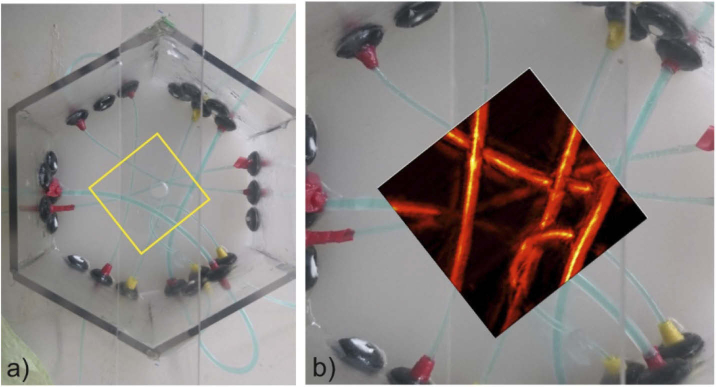
Measurement of intertwined tube structures: a) Tube phantom with ICG filled silicone tubes, before filling it with scattering agar. The yellow square illustrates the scan area of 30 × 30 mm, b) Maximum amplitude projection of the 3D Scan in z-direction superposed with the photograph.

Figure 9 shows a photographic top view of the silicon tubes of phantom 3, before it was filled with the scattering agar medium and the maximum amplitude projection of the tube phantom normal to the scan direction after reconstruction. In the experiment, the phantom was illuminated from the bottom. The scan area was 30 × 30 mm with 50 µm increment in x-direction and 200 µm in y-direction. The agreement of the reconstructed image with the tube phantom photograph demonstrates the imaging capability of more complex structures.

## 5. Discussion

The aim of this work was the development of a transducer for photoacoustic macroscopy that provides high sensitivity and lateral resolution over a large depth of field. In previous work on large DOF imaging with conventional lenses, image formation using synthetic aperture focusing from virtual detector positions [6] and model based reconstruction employing the sensor geometry were proposed [7]. These reconstruction-based methods overcome the limitation of the acoustic lens by using information contained in neighboring A-scans to generate a high-resolution image of out-of-focus regions. By contrast, the design proposed here uses an array of ring-shaped elements to reconstruct a large DOF A-scan that does not require information from neighboring scan positions. Furthermore, we use the concept of conical lenses, which are known to naturally produce an extended DOF. The combination of a low number of annular elements and its conical arrangement showed the required properties, both in simulations and in phantom experiments. In comparison with linear array based photoacoustic imaging devices, which also provide constantly high resolution over an extended depth range, the proposed small array uses much less complex electronics, offering a low-cost alternative in certain clinical applications.

An important property is the maximum imaging depth of the device. Due to the choice of relatively wide ring elements, to each ring a depth range can be assigned, where it reacts most sensitively to signals from small sources on the ring axis. This range is defined by the normal incidence of generated acoustic waves on the sensor area. The maximum depth is defined as the point on the axis, from which the wave arrives with normal incidence on the outermost point on the surface of the largest ring. With an inclination of 25° and a mean radius of 16.5 mm, the outermost ring has a theoretical depth limit of about 32 mm. It should be noted that after reconstruction an image of a source at this depth includes information from other rings, but with smaller amplitudes due to the oblique incidence. Furthermore, the reconstructed values are influenced by applying the coherence factor weighting for artefact reduction. At this point, the benefits of a combination with a flat ring element becomes apparent. The planar ring receives acoustic waves with normal incidence over the entire depth range. Its drawback is a low lateral resolution, which can be compensated by the reconstruction method. The experimental profile in Fig. 7 shows approximately constant amplitudes up to a depth of 32.5 mm, which agrees well with this estimate.

The above definition of the imaging depth does not consider the limitation due to the light attenuation, because it assumes equally strong acoustic sources over the entire depth range. To estimate a practical imaging depth, we used the results of the sensitivity measurement. With the aid of the NEP, the known illuminated surface and the corresponding pulse energy, a Monte Carlo simulation was used to determine the depth at which the initial pressure in the source reaches a value of 57 Pa. In the Monte Carlo simulation, optical parameters at 750 nm wavelength were used to simulate human breast tissue with a thin skin layer [29]. The illumination energy was set to the ANSI level of 25 mJ/cm^2^. Considering blood as the imaging target with a Grueneisen parameter of 0.16 [30] and an absorption coefficient of 0.4 mm^−1^ [29], a local fluence of  ∼ 8.9*10^−2^ mJ/cm^2^ is needed to cause a pressure of 57 Pa. This fluence value was reached in the Monte-Carlo simulation at a depth of 22.5 mm. This corresponds to the depth at which the obtained amplitude of the reconstructed A-scan equals to the noise level and thus depicts the maximum depth at which the signal of a point source would be detectable. It should be noted that this analysis based on Monte-Carlo simulation does not consider the influence of the acoustic attenuation.

A question remains about the optimum number of ring elements in the transducer. A better reduction of artefacts would have been achieved with a higher number of rings. However, the purpose of the current study was to develop a transducer that has a complexity comparable to single-element, focused detectors and therefore needs a minimum number of input channels in a data acquisition device. The effect of four rings in comparison with a single element transducer was demonstrated in the experiment for artefact reduction. The typical X-shaped artefacts arising in conical and ring sensor geometries could be greatly reduced with the help of dynamic focusing and coherence factor weighting, since each ring element causes an X with a different apex angle. However, some artefacts remain, which was also visible in the tube phantom measurement. Although all tubes were detected successfully and their shape in the reconstructed image agrees well with the photograph of the phantom, the remaining X-artefacts lead to a widening of the tubes in the maximum amplitude projection.

Further work on advancing the geometry of the sensor array, developing an improved reconstruction method as well as finding an improved shape and arrangement of the individual ring elements will therefore be necessary.

## 6. Conclusions

A photoacoustic macroscopy device is presented, which combines benefits of a conical sensor providing a constant resolution over a large depth of field and an array with its dynamic focusing capability. The manufacture of the ring array using electrically patterned PVDF film offers flexibility in the design of different transducer geometries. For the chosen geometry consisting of one planar and three inclined rings, we could demonstrate lateral and axial resolutions of 285 µm and 95 µm, respectively, within a DOF of 20 mm. The DOF was approximately equal to an estimate of the achievable imaging depth, based on the sensitivity of the array and optical properties of breast tissue. Compared to large arrays, as they are used in photoacoustic breast imaging, the data acquisition time for 3D volumes with the scanning array is much longer. However, data from a few neighboring scan positions are sufficient to utilize the full resolution of the array. Therefore, we believe that this kind of imaging device could be useful for diagnostics of limited regions of interest, for instance of suspicious lesions that have previously been identified with another imaging technique.
